# Genome Sequences Reveal Cryptic Speciation in the Human Pathogen *Histoplasma capsulatum*

**DOI:** 10.1128/mBio.01339-17

**Published:** 2017-12-05

**Authors:** Victoria E. Sepúlveda, Roberto Márquez, David A. Turissini, William E. Goldman, Daniel R. Matute

**Affiliations:** a Department of Microbiology and Immunology, School of Medicine, University of North Carolina, Chapel Hill, North Carolina, USA; b Department of Ecology and Evolution, University of Chicago, Chicago, Illinois, USA; c Biology Department, University of North Carolina, Chapel Hill, North Carolina, USA; University of California, Berkeley

**Keywords:** cryptic speciation, *Histoplasma*, phylogenetic species concept, genomic alignment, taxonomy

## Abstract

*Histoplasma capsulatum* is a pathogenic fungus that causes life-threatening lung infections. About 500,000 people are exposed to *H. capsulatum* each year in the United States, and over 60% of the U.S. population has been exposed to the fungus at some point in their life. We performed genome-wide population genetics and phylogenetic analyses with 30 *Histoplasma* isolates representing four recognized areas where histoplasmosis is endemic and show that the *Histoplasma* genus is composed of at least four species that are genetically isolated and rarely interbreed. Therefore, we propose a taxonomic rearrangement of the genus.

## IMPORTANCE

The evolutionary processes that give rise to new pathogen lineages are critical to our understanding of how they adapt to new environments and how frequently they exchange genes with each other. The fungal pathogen *Histoplasma capsulatum* provides opportunities to precisely test hypotheses about the origin of new genetic variation. We find that *H. capsulatum* is composed of at least four different cryptic species that differ genetically and also in virulence. These results have implications for the epidemiology of histoplasmosis because not all *Histoplasma* species are equivalent in their geographic range and ability to cause disease.

## INTRODUCTION

*Histoplasma capsulatum* is a dimorphic ascomycete fungus that causes histoplasmosis in humans. Originally discovered in Panama in 1906 by S. T. Darling (1), its morphology in infected tissues led to its original classification as a protozoan. After multiple reclassifications, this fungus was determined to be closely related to other pathogenic dimorphic fungi such as *Coccidioides*, *Blastomyces*, and *Paracoccidioides* (2, 3). The haploid nature of its genome, combined with a variety of plasmid-based molecular genetic tools, have made *H. capsulatum* the most developed genetic system among dimorphic fungi (4, 5).

Histoplasmosis, the disease caused by *H. capsulatum*, is arguably the most common fungal respiratory infection worldwide, with hundreds of thousands of new infections occurring annually in the United States alone (6, 7). The disease burden of pulmonary histoplasmosis is among the highest of any disease caused by a primary fungal pathogen (8–10). In 5 to 10% of cases, the infection progresses in the lung or disseminates to visceral organs and can be difficult to treat with antifungal drugs. Patients who appear to recover may suffer a recurrence months to years later, particularly if they are immunocompromised by chemotherapy (8), organ transplant (11), or AIDS (12, 13). In fact, up to 25% of AIDS patients living in regions where *Histoplasma* is endemic develop histoplasmosis and require prolonged antifungal therapy (14, 15). The potential for environmental exposure and individual reactivation ensures that histoplasmosis will persist as a public health problem: by age 20, more than 80% of individuals residing in areas of endemicity in the continental United States are skin test positive for a previous infection (7, 16).

Despite the importance of the disease and the development of *Histoplasma* as a genetic model system, many aspects regarding the natural history, evolution, and systematics of this pathogen remain largely unknown. The fungus is found in soil and is commonly associated with environments where guano and bird droppings are present (17–19). This association has led to the provocative hypothesis that flying animals might be involved in the dispersion of *Histoplasma* (17, 20). The fungus can also be found in soil (21–23) and mammalian reservoirs (24), but the extent of its natural occurrence remains largely unexplored (25, 26). Even though the fungus is known to produce a sexual stage under laboratory conditions (*Emmonsiella capsulata* [27]), this form has never been observed in nature. The magnitude of sexual recombination is thus still undetermined.

The number of species encompassed in the genus *Histoplasma* also remains unknown. Geographically, *Histoplasma* has the widest range of all the dimorphic fungi, being globally distributed (17, 28–30). There seems to be strong population structure across continents, which poses the possibility of undiscovered events of speciation (28, 31, 32); however, all populations of the genus are currently classified as *H. capsulatum*. Three distinct varieties, determined by geographic distribution, morphology, and clinical symptoms, have been historically recognized. This classification system subdivides *H. capsulatum* into New World human pathogens (*H. capsulatum* var. *capsulatum*), African human pathogens (*H. capsulatum* var. *duboisii*), and Old World horse pathogens (*H. capsulatum* var. *farciminosum*) (33, 34). Seminal studies revealed that these three classic groups are artificial. Instead, *H. capsulatum* is composed of multiple genetically distinct genetic groups (at least four) that until now have never been recognized as species (31). These genetically isolated groups have differences in virulence (35), and one of these groups (*H. capsulatum* var. *duboisii*, henceforth referred to as “Africa”) seems to cause a unique disease (36, 37). Notably, *H. capsulatum* var. *farciminosum* was found to be polyphyletic, which indicates the traditional methods to delimit *Histoplasma* varieties have significant shortcomings (31).

The identification of cryptic species and the delimitation of species boundaries in fungi have propelled the research required to identify differences in morphology, virulence strategies, drug susceptibility, and natural history that were initially not apparent (38, 39). In *Histoplasma*, some of these differences between genetic clusters have already been observed, ranging from extensive differences in virulence to drug susceptibility for multiple genetic clusters within the genus. The natural question is to assess whether these clusters are indeed isolated species or not. Disentangling the species limits within *Histoplasma* would be beneficial not only to understand the systematics and evolution of this group of pathogens. If the reported differences are ascribed to the existence of species within the group, then the identification of such species and of the genetic factors that make them unique might contribute to the identification of virulence strategies and the design of species-specific treatments.

In spite of the clear importance of defining species boundaries in pathogens, the task is not straightforward in fungi (reviewed in references 40 to 42). The biological species concept, the gold standard for animals and plants, states that species are groups of individuals that cannot interbreed in sexual crosses (38–43). However, delimiting species under this concept necessarily requires performing large-scale genetic crosses between individuals of potentially different species, which is challenging in eukaryotes such as fungi and almost impossible in *Histoplasma*, where sexual stages have not been observed in nature and crosses are formidably challenging to perform *in vitro*. In the absence of crosses, indirect measurements can be used to infer the occurrence of speciation in lieu of the ability to interbreed.

Genetic clusters that are discontinuous from each other can also be detected using DNA divergence. Several frameworks have been proposed to detect such discontinuities, but the phylogenetic species concept (PSC) is the most extensively used in fungi (reviewed in references 40, 41, and 43). The PSC has had several propositions over the years, but all of them are tailored to detect monophyletic and diagnosable groups that do not interbreed with each other (41, 44). The most practical and comprehensive test for the identification of phylogenetic species is to identify monophyletic groups with high levels of support, which might in turn reflect the existence of reproductively isolated groups (45–48). Dimorphic fungi are a natural group for the use of phylogenetic approaches to detect cryptic species because even though some of them can be crossed, the majority have no known sexual stage, which makes them unsuitable for the application of the biological species concept.

Here we test the hypothesis that *Histoplasma capsulatum* is composed of several monophyletic and identifiable species using whole-genome data. Population genetics and phylogenetic analyses indicate the existence of five isolated genetic lineages that are consistent with the existence of more than one species. Given the remarkable genealogical consistency across the whole genome, we propose that four of these genetic groups, all endemic to the Americas, should be elevated to species status. The fifth group, the African clade of *Histoplasma* (*H. capsulatum* var. *duboisii*, or “Africa”), is likely to be a separate species as well, but we refrain from modifying its taxonomic affinity without more sampling that allows us to reliably measure its within-group genetic diversity and quantify its level of differentiation from other *Histoplasma* species. Our approach provides a blueprint for the identification of species boundaries in fungi using whole-genome sequences. These results open the possibility of studying the biological barriers that lead to genetic discontinuities across fungal species but also to examine how much gene flow occurs between cryptic species of fungal pathogens.

## RESULTS

### Genomic data.

All genome sequences reported in this study have been deposited in the Short Read Archive, and intermediary processing files (.sam, and.bam) have been deposited in Dryad. The coverage for each reported genome is reported in Table S1 (at https://doi.org/10.5061/dryad.006bf). In spite of the differences in genome size (49), the coverage for all lines was over 40×, and in most cases, the levels of coverage were similar among species (one-way analysis of variance [ANOVA], *F*^4,26^ = 3.3822, *P* = 0.0235 [see Table S2 at https://doi.org/10.5061/dryad.006bf]). There were two exceptions. NAm 2 (i.e., North America 2) shows slightly lower coverage than NAm 1 (NAm 2 average coverage, 58.33; NAm 1 average coverage, 78.35; Tukey contrasts, *t*_26_ = −2.946, *P* = 0.0452) and LAm A (i.e., Latin America A) (LAm A average coverage, 83.18; Tukey contrasts, *t*_26_ = −2.917, *P* = 0.0482 [see Table S3 at https://doi.org/10.5061/dryad.006bf]). These results are consistent with NAm 2 having a larger genome than all other species (49).

### *Histoplasma* harbors at least five discrete genetic clusters.

**FIG 1:**
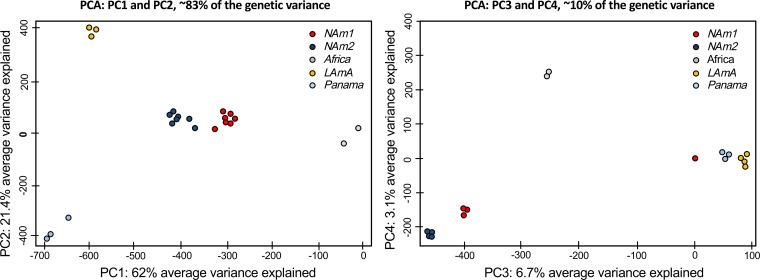
Principal-component analysis for *Histoplasma* using whole-genome allelic frequencies. The genetic variance in the genus *Histoplasma* indicates the existence of discrete genetic clusters. Red, NAm 1; blue, NAm 2; yellow, LAm A; gray, Africa; light blue, Panama. (Left panel) Principal components (PC) 1 and 2 are shown, and the percentage of variance explained by each eigenvalue is shown within parentheses on each axis. (Right panel) PC3 and PC4.

We employed a population genetics framework to quantitatively assess if the patterns of genetic variation were consistent with the existence of different species within *Histoplasma*. First, we calculated levels of within-species population variation. We did a principal-component analysis (PCA) to analyze allelic frequencies across the whole genome. The first five principal components (PC1 to -5) of the PCA explained about 95% of the genetic variance. The results suggested three genetic patterns (Fig. 1). The vast majority of variation is explained by the first four PCs. The first four PCs differentiate between species. PC1 (variance explained, 62%) clearly differentiates five genetic clusters within *Histoplasma*. These clusters are concordant with the groups reported by Kasuga et al. (31) and cluster according to geography. The differentiation between these genetic groups is large and complete as there is no overlap between isolates from different putative species. The NAm 1 and Africa groups are the most differentiated groups (in terms of their allele frequencies), and they appear to be highly differentiated along PC1. PC2 (variance explained, 21.4%) mainly differentiates between the Africa and Panama species, but all other species also appear differentiated. PC3 (~7%) separates the North American clade from the other three species, while PC4 (~3%) strongly differentiates between NAm 1 and NAm 2. No obvious patterns explained PC5 (explaining <2% of the variance), which might be related to variation within each species. These genotype analyses revealed that genetic variability in *Histoplasma* is portioned across genetic clusters and that such clusters seem to have little overlap in terms of allele frequencies. This is consistent with genetic differentiation and little gene flow between clusters. We thus explored whether these clusters fulfill the definition of a phylogenetic species and thus should be elevated in taxonomic status.

### The genetic clusters within *Histoplasma* are cryptic species.

We next used whole-genome data to reconstruct the genealogical relationships between the sequenced individuals of *Histoplasma*. We rooted the tree with two closely related species: *Emmonsia parva* and *Emmonsia crescens*. The tree reveals two sets of results: whether the genetic clusters are phylogenetic species and the phylogenetic relationships between such species.

### (i) Recognition of phylogenetic species.

**FIG 2:**
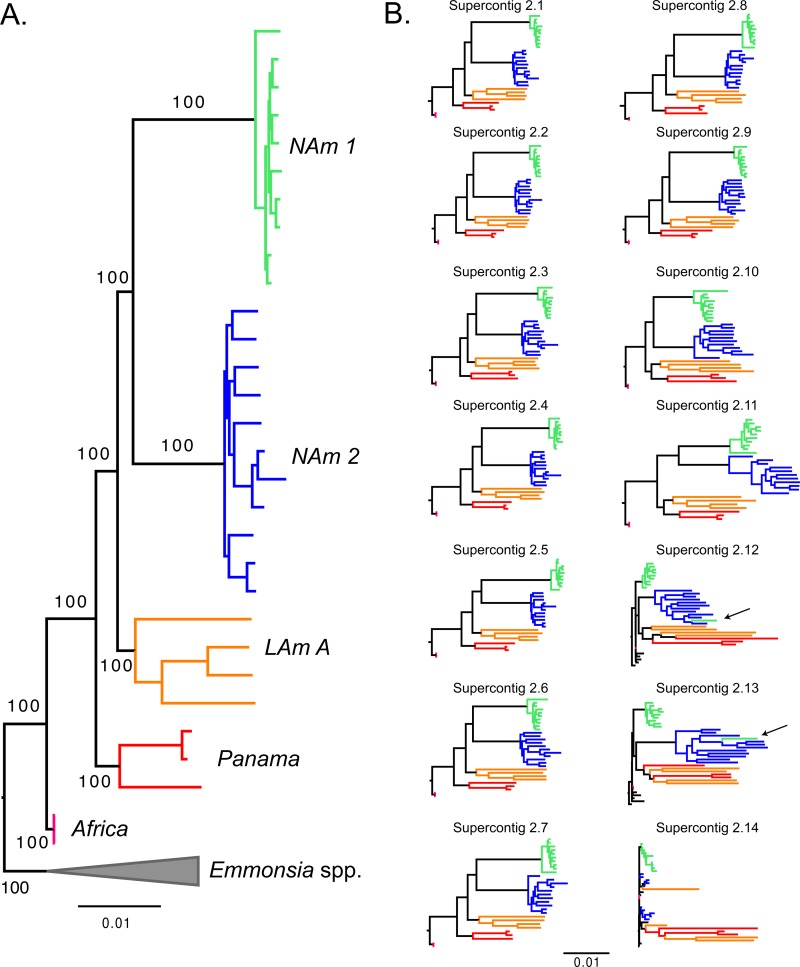
Rooted phylogram for the species of the *Histoplasma* genus using whole-genome data. The leftmost tree (A) shows a tree constructed using the variation from the whole genome. The trees to the right (B) were built with data from each of the 14 supercontigs. (The length of each supercontig is not shown). The length of each branch indicates the amount of genetic divergence. Arrows in the trees to the right show isolates with conflicting positioning for a given supercontig. All topologies are rooted with *Emmonsia* sp.

Our whole-genome concatenated phylogenetic reconstruction indicates *Histoplasma* is a highly genetically structured group. Congruent with the results from the PCA, five of the groups proposed by Kasuga et al. (the NAm 1, NAm 2, LAm A, Panama/H81 lineage, and Africa groups) (28, 31) form clades with high support (Fig. 2A). We next analyzed whether these groups fulfilled the requirements to be considered phylogenetic species (*sensu* [45, 50]). First, we found that all the genetic clusters are reciprocally monophyletic (i.e., all five groups coalesce within each other, before any coalescence events take place between groups), a primary requirement for species definition (51, 52). Second, we determined whether the five identifiable clades of *Histoplasma* had enough support to be classified as different phylogenetic species. In all five cases, the concordance factors were high enough to argue these clades are indeed different species (following reference 45). The support for the branches leading to each phylogenetic species is shown in Table S4 (at https://doi.org/10.5061/dryad.006bf). Single superscaffold analyses revealed a similar pattern. NAm 1 was monophyletic and strongly supported in all superscaffold trees, NAm 2 and *H. capsulatum* var. *duboisii* were supported by the 13 longest scaffolds, and all 5 groups were supported by the 11 longest scaffolds (Fig. 2B). The phylogenetic relationships between these groups in the superscaffold trees were identical to those observed in the concatenated tree in most cases, the exception being superscaffolds 10 to 13, where the Panama and LAm A groups were sister taxa, instead of sequential sister groups to the North American clade (NAm 1/NAm 2). It is worth noting these scaffolds are much smaller than the first 10 and thus contain fewer informative sites (see Table S5 at https://doi.org/10.5061/dryad.006bf). Collectively, the genetic differentiation, the reciprocal monophyly, and the high levels of support strongly suggest that the five genetic groups of *Histoplasma* are indeed phylogenetic species.

### (ii) Species tree.

**FIG 3:**
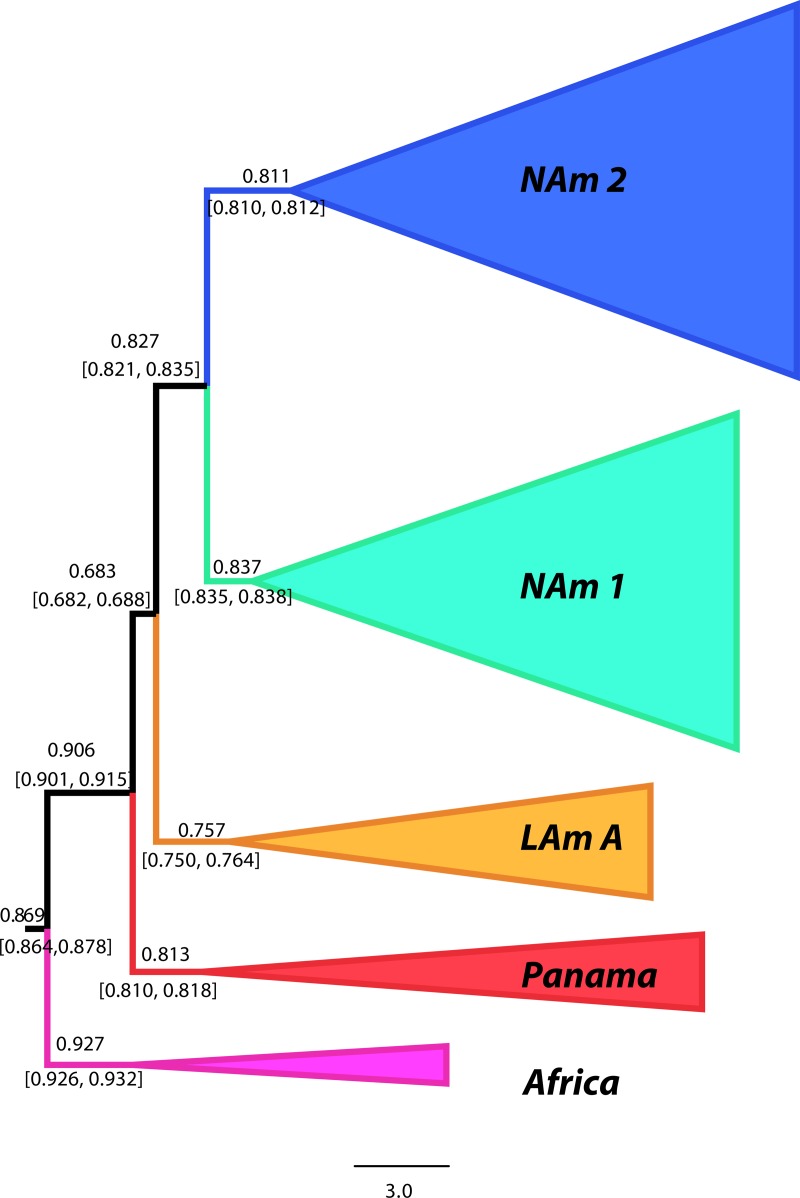
Species tree of the genus *Histoplasma* shows that all species have high levels of genomic concordance. Shown is a genome-wide primary concordance tree produced by BUCKy (α = 5), based on 100-kb windows. Values above each branch show the concordance factor (CF) for each node, and values below branches show the 95% Bayesian credibility intervals for this statistic.

Our phylogenetic analyses indicate a large level of concordance among genomic windows and at the whole-genome level (Fig. 2). We quantified the magnitude of the concordance and inferred the likely *Histoplasma* species tree with gene tree analysis using BUCKy (53). This method inferred an identical species tree topology to the topology produced by the RAxML analysis of the whole genome (Fig. 2A and 3). We used 100-kb windows as the unit for analysis because of the high linkage disequilibrium (LD) in all species of *Histoplasma* (i.e., *r*^2^ is on the order of 0.2 after 100 kb [see Fig. S1 in the supplemental material]). The nodes grouping isolates for each species had concordance factor (CF) values larger than 0.75, and the other nodes in the tree had values above 0.68 which indicates that the vast majority of the genome recapitulates the species tree and is in concordance with our proposed phylogenetic species. These levels of concordance are higher than those shown by *Neurospora* species (54), which in turn provides further support that the phylogenetic species of *Histoplasma* are not simply the result of population structure but are indeed truly isolated genetic clusters.

10.1128/mBio.01339-17.1 FIG S1  Linkage disequilibriums persist over large regions of the genome in *Histoplasma*. Average LD as measured by *r*^2^ between pairs of SNPs with distances binned every 100 bp. Download FIG S1, PDF file, 4.9 MB. Copyright © 2017 Sepúlveda et al. 2017 Sepúlveda et al. This content is distributed under the terms of the Creative Commons Attribution 4.0 International license.

### (iii) Phylogenetic relationships between groups.

We next assessed how the five phylogenetic species were related to each other. The tree topology is shown in Fig. 2A and 3. The 10 largest supercontigs, which account for 98.50% of the genome, also show the same genealogy (Fig. 2B). The relationship between these species was surprising because it strongly contradicts previously proposed relationships (20). First, and unlike previous reports, we find that all four American species are closely related and form a monophyletic group. The African specimens are an outgroup to the American group, suggesting a possible event of vicariance that led to the formation of the African and American clades. These results also indicate that several speciation events have occurred since *Histoplasma*’s arrival onto the American continent. These results seem to confirm the initial hypothesis of an American *Histoplasma* clade, initially dubbed *H. capsulatum* var. *capsulatum*, that is actually composed of at least four species (but see Discussion).

Notably, we find that the two North American species, NAm 1 and NAm 2, are sisters. This contradicts previous reports that had these two groups as highly divergent (20). A single isolate of the NAm 1 group (WU24) fell within the NAm 2 clade in supercontigs 10 to 13 (arrows in Fig. 2B), which might indicate these two species are still able to exchange genes. Alternatively, this may be due to low levels of incomplete lineage sorting (ILS). These alternatives are explored below. Collectively, these results constitute the first genome-wide approach to obtain resolution on the genealogical relationships between *Histoplasma* isolates.

The isolate MV3 collected in Colombia is part of the Panama lineage. This result expands the known range of the Panama species, initially thought to be restricted to Central America, to northern South America, which is part of the geographic range of LAm A (e.g., MZ5, also a Colombian isolate, is part of LAm A). This makes the Panama and LAm A groups sympatric. Just as it occurs with NAm 1 and NAm 2, this overlap is of particular importance to signal whether speciation has taken place because the two species pairs share a geographic range. This means that these two species have had the chance to interbreed; if no barriers to gene flow were in place, then their genomes would have undergone genome homogenization. Note that smaller supercontigs (2.12 to 2.14; less than 0.5% of the genome) show evidence of shared ancestry that might be caused by gene exchange or ILS circumscribed to these genomic regions. All these results are strongly suggestive of reproductive isolation between the Panama and LAm A groups and between NAm 1 and NAm 2.

### (iv) Genetic distance.

**FIG 4:**
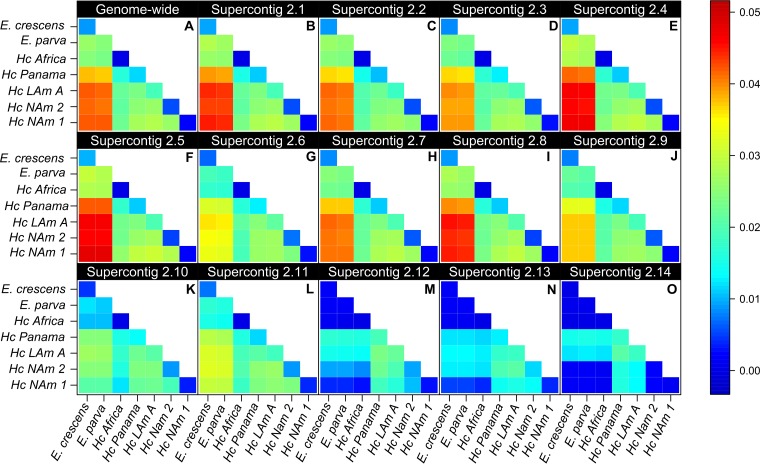
Pairwise genetic distance indicates that *Histoplasma* species are genetically differentiated across the whole genome. Values in the diagonal are the estimate of π_intra_ (intraspecific variability). All other values are π_inter_ (genetic distance between species). As expected *Emmonsia crescens* is more distantly related to *Histoplasma* species than they are to themselves. In all cases, π_inter_ is higher than π_intra_, indicating strong genetic differentiation (Table 1).

We measured the magnitude of genetic differentiation between species pairs. To this end, we next quantified π_inter_, a measurement of divergence among all putative species of *Histoplasma*, including the two sympatric pairs. By comparing the magnitude of between-species differentiation with within-species differentiation, one can assess the extent of differentiation and gene flow between putative species. We found that genetic variability is partitioned among species and that in all cases the magnitude of interspecific distances is at least 2× higher than that of intraspecific distances for all species pairs in genome-wide estimations (Fig. 4A; Table 1). In other words, the differentiation between species in all pairwise comparisons is larger than the amount of genetic variability within species. Notably, this pattern holds even though the sampling of some of the groups (e.g., the Africa and Panama groups) is rather modest. The 14 largest supercontigs show remarkably similar patterns, indicating that the genetic differentiation among species is pervasive across the genome (Fig. 4B to O). These results are in line with the PCA and the phylogenetic reconstruction and collectively suggest strong genetic differentiation among *Histoplasma* species.

**TABLE 1:** Genetic distances between species are at least two times larger than the amount of intraspecific variation in all pairwise comparisons among *Histoplasma* species^a^

Species 1	Species 2	π_intra1_	π_intra2_	π_inter_	*Z* value	*P* value
NAm 1	NAm 2	5.870 × 10^−3^	6.705 × 10^−3^	0.0243	11.767	<1 × 10^−4^
NAm 1	LAm A	5.870 × 10^−3^	1.246 × 10^−2^	0.0263	8.8614	<1 × 10^−4^
NAm 1	Panama	5.870 × 10^−3^	1.142 × 10^−2^	0.0254	8.5931	<1 × 10^−4^
NAm 1	Africa	5.870 × 10^−3^	3.290 × 10^−6^	0.0213	7.618	<1 × 10^−4^
NAm 2	LAm A	6.705 × 10^−3^	1.246 × 10^−2^	0.0282	8.1891	<1 × 10^−4^
NAm 2	Panama	6.705 × 10^−3^	1.142 × 10^−2^	0.0278	7.4744	<1 × 10^−4^
NAm 2	Africa	6.705 × 10^−3^	3.290 × 10^−6^	0.023	3.7805	<1 × 10^−4^
LAm A	Panama	1.246 × 10^−2^	1.142 × 10^−2^	0.0235	2.6809	6.00 × 10^4^
LAm A	Africa	1.246 × 10^−2^	3.290 × 10^−6^	0.0217	2.2539	0.0238
Panama	Africa	1.142 × 10^−2^	3.290 × 10^−6^	0.0163	1.8327	0.0404

a π_inter_ and π_intra_ (i.e., π_intra1_ and π_intra2_ pooled) were compared using two-sample Fisher-Pitman permutation tests. The significance of the *Z* value was assessed using 9,999 Monte Carlo resamplings.

### Approximate divergence between *Histoplasma* species pairs.

To assess whether any shared ancestry between species pairs (see immediately below) was most likely caused by incomplete lineage sorting, we calculated approximate divergence dates for four nodes in the tree: the node where NAm 1 and NAm 2 split, the node where (NAm 1/NAm 2) and LAm split, the node where [(NAm 1/NAm 2), LAm] and the Panama group split, and the node where {[(Nam 1/NAm 2), LAm A], Panama group} and the Africa group split. The approximate divergence times are shown in Table 2 and indicate that all speciation events are over 1.7 million years. We converted these divergence times to generations assuming that *Histoplasma* has 1, 10, or 100 generations per year and calculated the time required to lose an allele segregating in an ancestral population. These calculations are shown in Fig. S2 in the supplemental material. In all cases, and regardless of the assumptions regarding the number of generations per year or effective population size, the time to lose a neutral polymorphic allele in the ancestral species is much smaller than the divergence time between any of the species pairs of *Histoplasma*. (None of these calculations incorporates the possibility of trans-specific balancing selection.) These results suggest that any shared ancestry among *Histoplasma* species is most likely caused by introgression and not by incomplete lineage sorting.

10.1128/mBio.01339-17.2 FIG S2  Identified introgressions between *Histoplasma* species are unlikely to represent ancestral variation that is still segregating in the recipient species. Each panel shows the expected number of generations that an allele at a given frequency, *p*, would take to either be fixed (black line) or lost (red line) from the population. Horizontal lines denote the number of populations since the two species diverged, assuming 1 generation per year (solid blue line), 10 generations per year (dashed blue line), or 100 generations per year (dotted blue line). (A) *N*_*e*_ = 10^6^. (B) *N*_*e*_ = 10^5^. (C) *N*_*e*_ = 10^4^. Note *y* axes are in log scale. Download FIG S2, PDF file, 0.03 MB. Copyright © 2017 Sepúlveda et al. 2017 Sepúlveda et al. This content is distributed under the terms of the Creative Commons Attribution 4.0 International license.

**TABLE 2:** Approximate divergence times for four nodes in the tree shown in species pairs of *Histoplasma*

Node/speciation event	MYA (CI)^a^	No. of generations since speciation (*G*)^b^:		
		1 GPY	10 GPY	100 GPY
NAm 1/NAm 2	1.750 (1.37–2.12)	1.75 × 10^6^	1.75 × 10^5^	1.75 × 10^4^
(NAm 1/NAm 2), LAm	2.010 (1.76–2.26)	2.01 × 10^6^	2.01 × 10^5^	2.01 × 10^4^
[(NAm 1/NAm 2), LAm], Panama	2.110 (1.99–2.23)	2.11 × 10^6^	2.11 × 10^5^	2.11 × 10^4^
{[(NAm 1/NAm 2), LAm], Panama}, Africa	2.400 (2.19–2.61)	2.40 × 10^6^	2.40 × 10^5^	2.40 × 10^4^

a MYA, million years ago; CI, confidence interval.

b *G*, expected number of generations. We assumed three different numbers of generations per year (GPY) to convert from divergence time to number of generations.

### The cryptic species of *Histoplasma* exchange genes rarely.

Species are genetic clusters discontinuous from each other. We tested whether the putative species of *Histoplasma* exchange genes with each other using three different approaches: (i) assessing the most likely number of admixture events in the tree using TreeMix, (ii) calculating the amount of introgression in different species pairs, and finally (iii) identifying potentially admixed individuals.

### (i) Admixture events.

**FIG 5:**
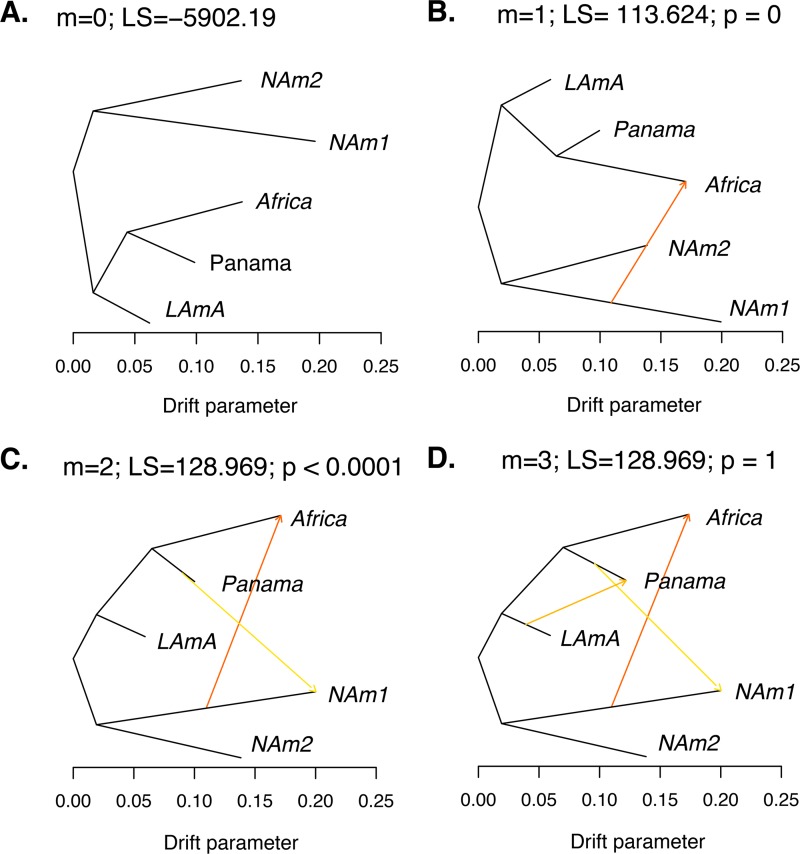
TreeMix results show genetic differentiation between species from the *Histoplasma* genus. (A) Best-fitting genealogy without admixture for *Histoplasma* species calculated from the variance-covariance matrix of genome-wide allele frequencies. We estimated the likelihood of three demographic scenarios with 1 to 3 migration events (*m*). The caption for each panel shows the number of migrations (*m*), the LS of the demographic scenario, and the *P* value (p) of the comparison between that model and the model with m^−1^ migration events (also shown in Table 3). (B) *m* = 1. (C) *m* = 2. (D) *m* = 3. To determine the best-fitting demographic scenario, we used LRTs to compare nested models (one migration versus two migrations and two migrations versus three migrations). We also used wAIC to find the best-supported migration scenario (see text). The caption for each panel contains the number of migrations, the likelihood, and the associated *P* value when comparing that demographic scenario with the previous one. The best-fitting demographic history involved two migration events: one from NAm 1 to Africa and a second one from Panama to NAm 1 (B).

We measured the extent of gene exchange between phylogenetic species. To this end, we used the program TreeMix to identify instances of gene flow between *Histoplasma* species. TreeMix uses deviations from the genetic covariance predicted by a tree-like pattern of diversification to identify large events of gene exchange between species. This unrooted genealogy without accounting for gene flow (Fig. 5A) perfectly corresponds to our phylogenetic trees. We next sequentially added migration events and assessed whether they improved the likelihood of the tree. We found two migration edges were the most likely mixture scenario with both edges involving the NAm 1 branch (Fig. 5C). The most likely migration edge indicates the existence of a gene exchange event from NAm 1 to Africa (Fig. 5B and C; weight, 0.451). The second edge shows a milder migration event and indicates the migration of genetic material from Panama to NAm 1 (Fig. 5C; weight, 0.234). We inferred the same scenario using a likelihood ratio test (LRT) to assess the most likely number of migration edges or by weighted Akaike information criterion (wAIC [one migration edge versus two migration edges]) (Fig. 5B and C; Table 3). Addition of an additional migration event did not improve the log likelihood (two migration edges versus three migration edges) (Fig. 5D; Table 3). The drift parameter, which is proportional to the age of the migration, revealed that the two genetic exchange events are unlikely to be recent.

**TABLE 3:** A demographic scenario that involves two migration events is the most likely explanation for the partition of allele frequencies across the genus *Histoplasma*^a^

*m*	LS	LRT	*P* value	AIC	wAIC
0	−5,902.19			11,804.38	2.431 × 10^−49^
1	−5,845.378	113.624	0	11,692.76	4.205 × 10^−25^
2	−5,788.566	128.969	2.17 × 10^−7^	11,581.13	0.731
3	−5,788.566	0	1	11,583.13	0.269

a As shown in Fig. 5C. All calculations were done with the model implemented in the package TreeMix. *m*, number of migration edges; LS, likelihood score; LRT, likelihood ratio test performed with the model that involved one fewer migration event; *P* value, level of significance of the LRT with the previous model (i.e., *m* versus *m*^−1^) using χ^2^ with 1 df; AIC, Akaike information criterion; wAIC, weighted AIC for each model.

### (ii) Potential introgression.

We corroborated the results from TreeMix by assessing occurrence of ancestral admixture using the ABBA/BABA D statistic. This approach also rules out the possibility that shared ancestry is caused by incomplete lineage sorting. We tested three hypotheses: (i) that there has been introgression between NAm 1 and Africa, (ii) that there has been migration between NAm 2 and Panama, and (iii) that there has been introgression between NAm 1 and NAm 2. The reasoning for the first two migration events was the strong suggestion of gene exchange from TreeMix, while the third event was tested because these are the most closely related species in the genus and also the ones that cooccur. All these events were corroborated with the D statistic, which suggests introgression between these three species pairs (Table 4). Notably, the D statistic detects introgression between NAm 1 and NAm 2, which was not detected by TreeMix. The reasons for this incongruence remain unknown but might be related to the larger sample size of NAm 1 and NAm 2, which might reveal introgressions at low allele frequency, which are not detected by TreeMix.

**TABLE 4:** D statistic variations and *f*_*d*_ show evidence for admixture between three species pairs of *Histoplasma*^a^

Allopatric population	Recipient population	Donor population	Outgroup	D	*Z* score^b^	*f_d_*^c^
NAm 2	NAm 1	Panama	*Emmonsia parva*	0.187	505.602	0.036
NAm 1	Africa	NAm 1	*E. parva*	0.198	500.946	0.043
LAm A	NAm 1	NAm 2	*E. parva*	0.186	527.217	0.040

a TreeMix had provided evidence for two of the species pairs (NAm 1/Africa and NAm 1/Panama). The third pair (NAm 1/NAm 2) is a syntopic pair.

b For details, see references 103 and 104.

c For details, see reference 104.

We next used the *f*_*d*_ statistic (55) to estimate the proportion of the donor genome that had introgressed from recipient in the three gene exchange scenarios described above. We found that on average, the proportion of the genome that has been introgressed across species boundaries is very small (less than 5% of the genome [Table 4]). This proportion was roughly equivalent regardless of the used outgroup. More work and a fine dissection of the precise allele frequency and haplotype size will be required to assess whether these low levels of introgression are caused by low allele frequencies (i.e., a few individuals are admixed), by small haplotypes (i.e., introgression might be common but small), or both.

### (iii) Admixed individuals.

**FIG 6:**
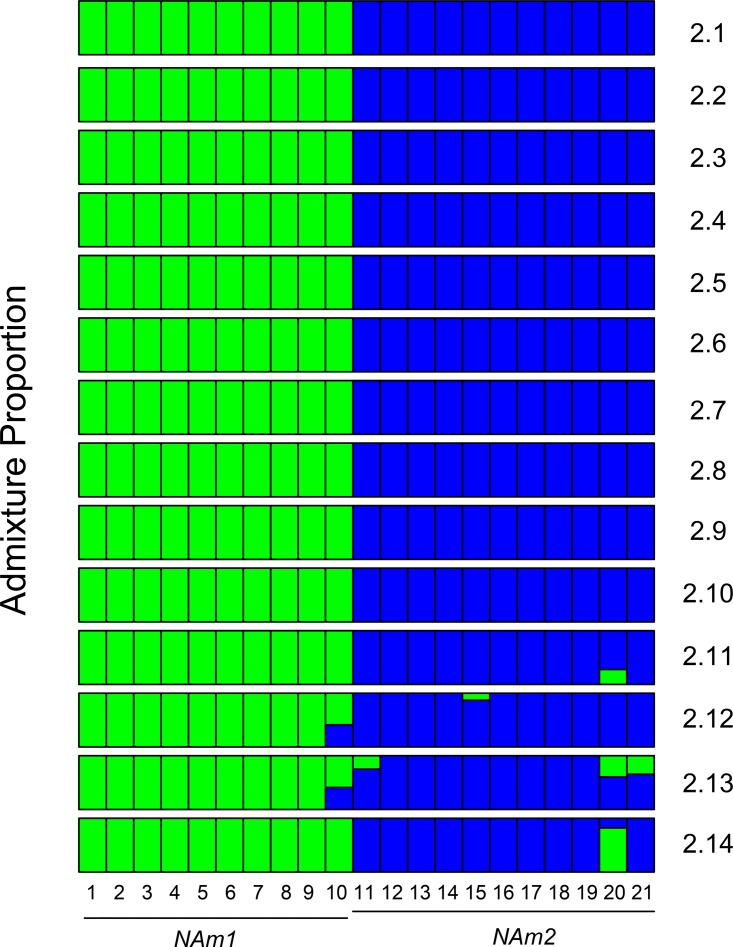
ADMIXTURE results for NAm 1 and NAm 2. ADMIXTURE proportions among NAm 1 and NAm 2 isolates (*K* = 2). Each of the two clusters is represented by a different color, and each isolate is represented by a vertical line divided into two colored segments with heights proportional to genotype memberships in the clusters. Thin black lines separate isolates. 1: 505; 2, CI_19; 3, CI_22; 4, CI_24; 5, CI_42; 6, CI_43; 7, CI_7; 8, Downs; 9, UCLA_531; 10, WU24; 11, 1986; 12, CI_10; 13, CI_17; 14, CI_18; 15, CI_30; 16, CI_35; 17, CI_4; 18, CI_6; 19, CI_9; 20, G217B; 21, G222B. We ran independent analyses for the largest 14 supercontigs, indicated by the number on the right. Results from the whole-genome analyses are concordant with the results from supercontigs 2.1 to 2.10.

Finally, we assessed whether we could detect specific individuals with mixed ancestry. We focused on the most closely related species pair, which was also the pair with the largest population sample individuals: NAm 1/NAm 2. (The currently small sizes and deep divergence between conspecific isolates of the Africa, Panama, and LAmA samples preclude the possibility of this analysis.) We used ADMIXTURE to identify if any of the individuals from these two species showed evidence of being hybrids. The results from this analysis are largely congruent with the findings from our phylogenetic analyses. WU24, a NAm 1 isolate, showed conflicting positions in the genealogies from different supercontigs (supercontigs 2.12 and 2.13). This isolate also show evidence of being admixed in the ADMIXTURE analysis (Fig. 6). Notably, ADMIXTURE indicates the possibility of other admixed individuals in NAm 2. 1986, CI30, and G222B show possible admixture in one of the smaller supercontigs (2.11, 2.12, 2.13, or 2.14). G217B shows evidence of admixture in supercontigs 2.11, 2.13, and 2.14.

Taken together, our results indicate that our putative species of *Histoplasma* represent genetically distinguishable, independently evolving lineages that seldom exchange genes, in some cases even while coexisting in sympatry. They should, therefore be elevated to species status.

### Systematics.

Based on our results, we propose to restrict the name *H. capsulatum* to the Panamanian lineage, as this species was originally described in the Canal Zone (1), and describe three additional species, corresponding to the two North American and South American lineages. Our results also indicate that *H. capsulatum* var. *duboisii* (Africa) is probably a separate species from *H. capsulatum*. However, given that our sampling is limited to two clonal isolates, we refrain from modifying this variety’s taxonomic status for the sake of taxonomic stability. Wider and more systematic strain sampling and exploration of its genetic diversity need to be achieved before confidently defining its taxonomic status.

### (i) *Histoplasma capsulatum sensu stricto* Darling 1906.

*Histoplasma capsulatum sensu stricto* Darling 1906. Mycobank accession number to be determined (TBD). Formerly known as the Panama or H81 lineage (31). Mycelial colonies on *Histoplasma*-macrophage medium (HMM) are white and cottony. Yeast colonies are variable in size, cream-colored (white to brown) on HMM agar. Yeast colonies are rough due to the presence of α-(1,3)-glucan in their cell wall. *Histoplasma capsulatum* can be discriminated from other *Histoplasma* species by PCR amplification, restriction fragment length polymorphism (RFLP) (mitochondrial DNA [mtDNA] and *ysp3*) (56), and randomly amplified polymorphic DNA (RAPD) (57, 58). The DNA fragment of α-tubulin amplified with the forward primer 5′-GGTGGCCAAATCGCAAACTC-3′ and reverse primer 5′-GGCAGCTTTCCGTTCCTCAGT-3′ (31) harbors 4 diagnostic single nucleotide polymorphisms (SNPs) of *H. capsulatum* (positions 1328, 1364, 1469, and 1575). *Histoplasma capsulatum* also has one diagnostic SNP at ADP-ribosylation factor (position 10; forward primer 5′-AGAATATGGGGCAAAAAGGA-3′ and reverse primer 5′-CGCAATTCATCTTCGTTGAG-3′), one at the H antigen precursor (position 710; forward primer 5′-CGCAGTCACCTCCATACTATC-3′ and reverse primer 5′-GCGCCGACATTAACCC-3′), and one at delta-9 fatty acid desaturase (position 1134; forward primer 5′ TTTAAACGAAGCCCCCACGG 3′ and reverse primer 5′-CACCACCTCCAACAGCAGCA-3′). *Histoplasma capsulatum* shows differential virulence in *in vitro* (macrophage) and *in vivo* (murine model [35]) assays compared to the two North American species.

Holotype: strain G186A. Isolated from a human patient in Panama in 1967 or before (31, 59, 60).

Etymology: We propose to restrict the use of *H. capsulatum* for the Panama group, as the organism was first discovered and described in Panama (1).

### (ii) *Histoplasma mississippiense* sp. nov.

*Histoplasma mississippiense* sp. nov. (MycoBank accession number MB823360). Formerly known as NAm 1. Mycelial colonies on HMM are white and cottony. Yeast colonies are variable in size, cream-colored (white to brown) on HMM agar. Yeast colonies are rough due to the presence of α-(1,3)-glucan in their cell wall. No sexual stage is known. The DNA fragment of α-tubulin amplified with the forward primer 5′-GGTGGCCAAATCGCAAACTC-3′ and reverse primer 5′-GGCAGCTTTCCGTTCCTCAGT-3′ (28) harbors 7 diagnostic SNPs of *H. mississippiense* (positions 1385, 1415, 1426, 1447, 1506, 1532, and 1556). *Histoplasma mississippiense* also has three diagnostic SNPs at ADP-ribosylation factor (positions 126, 235, and 310; forward primer 5′-AGAATATGGGGCAAAAAGGA-3′ and reverse primer 5′-CGCAATTCATCTTCGTTGAG-3′), four at the H antigen precursor (positions 504, 620, 640, and 740; forward primer 5′-CGCAGTCACCT CCATACTATC-3′ and reverse primer 5′-GCGCCGACATTAACCC-3′), and five at delta-9 fatty acid desaturase (positions 943, 1080, 1193, 1195, and 1206; forward primer 5′-TTTAAACGAAGCCCCCACGG-3′ and reverse primer 5′-CACCACCTCCAACAGCAGCA-3′) (28). Additionally, *H. mississippiense* can be discriminated from other *Histoplasma* species by RFLP (mtDNA and *ysp3* [58]) and RAPD (57). *Histoplasma mississippiense* also uses distinctive chymotrypsin-like serine proteases, which facilitates extracellular proteolytic activity (61). These enzymes are not present in any other *Histoplasma* species and are a diagnostic trait for *H. mississippiense*.

Holotype: Downs strain. Isolated in 1968 from an octogenarian woman with disseminated disease. Ex-type culture is preserved in the American Type Culture Collection (ATCC 38904).

Etymology: *mississippiense*; refers to the distribution of the species in the North American continent and the high density of its occurrence in the Mississippi Valley (7).

### (iii) *Histoplasma ohiense* sp. nov.

*Histoplasma ohiense* sp. nov. (MycoBank accession number MB823361). Formerly known as NAm 2. Mycelial colonies on HMM are white and cottony. Yeast colonies are variable in size, cream-colored (white to brown) on HMM agar. Yeast colonies are smooth due to the lack of α-(1,3)-glucan in their cell wall. No sexual stage is known. The DNA fragment of H antigen precursor amplified with the forward primer 5′-CGCAGTCACCTCCATACTATC-3′ and reverse primer 5′-GCGCCGACATTAACCC-3′ harbors seven diagnostic SNPs of *H. ohiense* (positions 631, 648, 654, 758, 782, 814, and 837). *Histoplasma ohiense* also has four diagnostic SNPs at ADP-ribosylation factor (positions 917, 942, 1026, and 1200; forward primer 5′-AGAATATGGGGCAAAAAGGA-3′ and reverse primer 5′-CGCAATTCATCTTCGTTGAG-3′), three at α-tubulin (positions 1369, 1473, and 1493; forward primer 5′-GGTGGCCAAATCGCAAACTC-3′ and reverse primer 5′-GGCAGCTTTCCGTTCCTCAGT-3′), and four at delta-9 fatty acid desaturase (positions 917, 942, 1037, and 1206; forward primer 5′-TTTAAACGAAGCCCCCACGG 3′ and reverse primer 5′-CACCACCTCCAACAGCAGCA-3′) (28). Additionally, *H. ohiense* can be discriminated from other *Histoplasma* species by RFLP (mtDNA and *ysp3* [56]) and RAPD (57, 58). Multiple studies have shown that *Histoplasma ohiense* is much more virulent than its sister species *Histoplasma mississippiense* (35). *Histoplasma ohiense* strains, unlike other *Histoplasma* species, have a smooth colony morphology and are virulent despite the lack of α-(1,3)-glucan in their cell wall. α-(1,3)-Glucan is a well-studied virulence factor in *H. capsulatum* that gives colonies a rough morphology. NAm 2 uses *Yps3p*, a homologue of the *Blastomyces dermatitidis* adhesin Bad1p, as a yeast-specific virulence factor. Notably, *Yps3p* is only essential for virulence in *H. ohiense* and not in the other species (62).

Holotype: strain G217B. Isolated in Louisiana, United States, in 1973 or before (31, 59, 60), from a human patient. Ex-type culture is preserved in the American Type Culture Collection (ATCC 26032).

Etymology: *ohiense*; refers to the distribution of the species in the North American continent and the high prevalence of the disease it causes in the Ohio Valley (8).

### (iv) *Histoplasma suramericanum* sp. nov.

*Histoplasma suramericanum* sp. nov. (MycoBank accession number MB823362). Formerly known as LAm A.

*Histoplasma suramericanum* can be discriminated from other cryptic species of *Histoplasma* by PCR amplification. The DNA fragment of H antigen precursor amplified with the forward primer 5′-CGCAGTCACCTCCATACTATC 3′ and reverse primer 5′-GCGCCGACATTAACCC-3′ (28) harbors three diagnostic SNPs of *H. suramericanum* (positions 591, 622, and 716). *Histoplasma suramericanum* also has one diagnostic SNP at ADP-ribosylation factor (position 154; forward primer 5′-AGAATATGGGGCAAAAAGGA-3′ and reverse primer 5′-CGCAATTCATCTTCGTTGAG-3′), and one at delta-9 fatty acid desaturase (position 1276; forward primer 5′-TTTAAACGAAGCCCCCACGG-3′ and reverse primer 5′-CACCACCTCCAACAGCAGCA-3′). Unlike *H. mississippiense* and *H. ohiense*, which cause a chronic pulmonary disease, *Histoplasma suramericanum* causes an acute pulmonary disease that leads to a higher patient mortality (63). *Histoplasma suramericanum* also causes a more pronounced lung pathology than *H. ohiense* (63).

Holotype: strain MZ5. Strain isolated in the Department of Antioquia (Colombia) in 2010 (Corporación para Investigaciones Biologicas [CIB] no. 15297).

Etymology: *suramericanum*; refers to the distribution of the species on the South American continent (31).

## DISCUSSION

### Cryptic speciation in *Histoplasma.*

Previous studies have suggested extensive differentiation among genetic clusters of *Histoplasma* (28, 31). Up to 15 clusters have been proposed, but no taxonomic changes have been made, as these reports were based on a handful of loci. In particular, Kasuga et al. (31) reported the existence of strong genetic discontinuities in *Histoplasma*. To date, this has remained the most systematic effort to understand the partition of genetic variation in *Histoplasma*, proposing seven phylogenetic species. Such efforts were seminal to set the stage to eventually identify cryptic species in *Histoplasma* but fell short for two reasons. First, they identified genetic groups but left “orphan” isolates that did not belong to any phylogenetic species, thus making *H. capsulatum* a polyphyletic group. This is problematic because under a phylogenetic species recognition framework, all individuals must belong to monophyletic groups (40, 41). Second, the limited genetic sampling (4 coding loci) provided only a partial portrait of the amount of genealogical diversity and discordance across *Histoplasma*. Using whole-genome sequences of 30 *Histoplasma* isolates from five of the seven phylogenetic groups found by Kasuga et al. (31), we find enough phylogenetic structure and genetic differentiation between these clusters to consider them separate phylogenetic species. Moreover, our analyses suggest that some hybridization and gene flow has occurred between species, but the magnitude of gene exchange is too small to preclude species boundaries.

The recognition of phylogenetic species is not devoid of caveats, as it does not explicitly assess the extent of reproductive incompatibility between species. It is possible that putative phylogenetic species actually represent genetic clusters formed by extrinsic (e.g., geographic) isolation, but which are still reproductively compatible. This issue might be the main limitation in the description of phylogenetic species; differentiation between cryptic speciation and local population structure is an issue especially pronounced when only a few loci are used (64, 65). It could be argued our approach might have a similar caveat because it is tailored to detect groups of isolated isolates using molecular markers. Yet, one would not expect congruent genealogies, reciprocal monophyly, and low levels of gene flow in locally structured populations at a genome-wide level (65, 66), and that is why our phylogenomic approach is superior.

Previous reports have evaluated the robustness of the criteria to identify phylogenetic species in fungi and have found that phylogenetic species recognition also predicts the boundaries dictated by the biological species concept. Yet, the origin of reproductive isolation is taxon specific, and a formal test of the suitability of the biological species concept in *Histoplasma* is still to be performed. However, we can unambiguously discard this scenario for at least one of the species pairs, *H. mississippiense*/*H. ohiense*, as their geographic distributions are largely overlapping, and they have been found in syntopy (28, 31). The existence of strong genetic differentiation despite the possibility to interbreed, facilitated by geographic and epidemiological overlap, indicates that these two lineages have speciated, since, in the absence of intrinsic reproductive isolation the observed genetic structure would have already broken down. This may also be the case for *H. suramericanum* and *H. capsulatum*, as their distributions overlap in central and possibly northwestern Colombia. However, no hybrid zones have been reported, although this could be due to sparse sampling of potential contact zones. The observation that most individual, unlinked, supercontigs, as well as 100-kb genomic windows, strongly support our proposed species delimitation further adds to the idea that the different genetic clusters within *Histoplasma* are not the result of early population structure but strongly imply that diversification in *Histoplasma* is at an advanced stage of divergence, probably past the point of speciation.

### Shared ancestry between species of *Histoplasma.*

Speciation is an evolutionary process that facilitates the segregation of genetic variation between species and makes genetic clusters differentiated from others. We found evidence of small levels of shared ancestry in three species pairs of *Histoplasma*. Two possible scenarios might explain this shared genetic material. First, introgression (i.e., the genetic movement of alleles across species boundaries) through the formation of hybrids (through interspecific crossings or somatic fusion) might be a cause for the sharing of alleles. Meta-analyses have estimated that up to 10% of animal and plant species might be able to hybridize (67). The extent of hybridization and introgression remains unknown for fungi. A second possibility is that alleles that segregate in both species predate speciation (50). This phenomenon known as incomplete lineage sorting occurs because reciprocal monophyly across the genome is not an instantaneous process and ancestral alleles might remain postspeciation. Three of our results indicate that there is little chance of ancestral polymorphism across species of *Histoplasma* and strongly suggest the possibility of low rates of introgression. First, the age of divergence of each of the species pairs indicates that incomplete lineage sorting is an unlikely event. The most recent speciation event in *Histoplasma* is still old; regardless of the assumptions of the time between recombination events, NAm 1 and NAm 2 diverged 1.7 million years ago. This old divergence suggests there is little chance for segregating ancestral polymorphism in the genome of these two sister species. (The likelihood of segregating ancestral polymorphism between any of the other species is even lower.) The second line of evidence is that the vast majority of detectable shared ancestry colocalizes to the same regions in the genome, which might suggest that shared SNPs are clustered in haplotypes possibly caused by introgression. Finally, the results from our D statistic analyses argue against this possibility and are consistent with introgression. Nonetheless, the testing of this hypothesis will need to wait until shared alleles are finely mapped in the genome to identify the size of the shared haplotypes as potential introgressions should show larger haplotype sizes than ancestral polymorphisms. More widespread sampling is sorely needed before we can assess the possibility of shared ancestry and its causes among other *Histoplasma* species.

### The diversification of the genus *Histoplasma*.

Our results also have important implications for the understanding of how fungal pathogens evolve. First, they contest the most recent available biogeographical hypothesis on the diversification of *Histoplasma* and the potential drivers of diversification in fungal pathogens. Teixeira et al. (20) argued that *Histoplasma* has experienced an accelerated rate of diversification in the last 4 million years, which might have been aided by dispersion of propagules by bats (also explored in references 17 and 68. Even though such a radiation—and the role of bats on it—remains a possibility that cannot be ruled out, our results lend weak evidence for this assertion. The phylogenetic relationships between *Histoplasma* spp. inferred from this multilocus sequence typing effort are not congruent with the phylogenomic information shown here. This type of inconsistency has been observed in other clades in which multilocus sequence typing can lead to incorrect phylogenetic relationships (65). Such a large overhaul of the understanding of the phylogenetic relationships among *Histoplasma* isolates renders all phylogenetic inference based on the erroneous phylogeny invalid.

The phylogenetic tree we produced also suggests the existence of a clade composed exclusively by American species of *Histoplasma*, with an African clade diverging early. It is premature to argue for (or against) the occurrence of an adaptive radiation after the colonization of the American continent. Before such hypothesis can be formally evaluated, it will be necessary to include isolates from a second Latin American group (LAm B) and evaluate the possibility of additional cryptic species within the clade. Moreover, samples of all the other localities where *Histoplasma* has been isolated (e.g., Eurasia, Australia, and Africa) are sorely needed to assess rates of diversification in *Histoplasma*. In general, a more extensive isolate collection will allow for testing the tempo and mode of evolution in the *Histoplasma* genus.

These results (and previous hypotheses) beg the question of what are the actual factors that have led to diversification in the *Histoplasma* genus. Dispersion by bats has been hypothesized to increase the geographic range of fungal pathogens and thus increase the likelihood of allopatric speciation. A robust analysis of coevolution between mammal reservoirs and different *Histoplasma* species would allow to quantify net diversification rates (speciation and extinction) and establish what have been the biological features leading to the existence of multiple species of *Histoplasma* and perhaps multiple virulence strategies in the genus. The current sampling in *Histoplasma* is too premature to perform this type of analyses, but our results provide a clear direction to identify the factors that underlie diversification in fungal human pathogens.

### Health implications.

These results also have epidemiological implications, as genomic differentiation among *Histoplasma* species could lead to differences in important disease traits. The differences in virulence and drug resistance among species seem to confirm this notion. For example, one of the North American species (*H. mississippiense*) is less virulent than the others (35, 58) but is more resistant to regularly used antifungals (69) and shows unique extracellular proteolytic activity (61). Another example is α-(1,3)-glucan, a well-studied virulence factor in dimorphic fungi that allows *Histoplasma* to escape host immune responses (70, 71). Despite the fact that the other North American species, *H. ohiense*, lacks α-(1,3)-glucan (present in the rest of the *Histoplasma* species), it is still virulent and is optimized for greater virulence in high-dose infections (35). The absence of α-(1,3)-glucan in *H. ohiense* strains could have selected for additional adaptations, and coevolution of alternative virulence factors may have arisen to compensate for the lack of this polysaccharide; e.g., *Yps3p*, a homologue of the *B. dermatitidis* adhesin gene *Bad1p* (72), is a virulence factor restricted to *H. ohiense* (61, 73). Future studies should address the precise rate of gene exchange across species. Even if these events of gene exchange are rare, they deserve to be evaluated closely. Admixture might serve as a mechanism to generate pathogens with new trait combinations that could pose an additional threat to human health.

A special case is that of *H. capsulatum* var. *duboisii*, also known as Africa. This group is highly differentiated from all the American species of *Histoplasma*. Additionally, this clade has been associated not with a lung disease, but with granulomatous lesions predominantly in subcutaneous, cutaneous, and osseous tissue (36, 74). Even though this epidemiological assessment might be premature as there have been no controlled infection experiments to compare disease caused by all five species, the occurrence of a new type of disease would indicate the evolution of a new virulence strategy within the genus *Histoplasma*. Moreover, we present evidence that even though this group is highly differentiated from all American *Histoplasma* species, Africa (*H. capsulatum* var. *duboisii*) might rarely exchange genes with *H. mississippiense*. This strong genetic differentiation and the ability to rarely exchange genetic material might in turn facilitate the transmission of virulence syndromes across species boundaries and across large geographic ranges. We do not propose a formal taxonomic elevation of this group to species as we only collected data for two isolates. We do expect that a systematic sample collection of Africa (*H. capsulatum* var. *duboisii*) will lead to the formal description of a new species.

### Conclusions and future directions.

Systematic explorations of phenotypic differences have revealed that *Histoplasma* encompasses a large phenotypic differentiation in terms of virulence, morphology, and pathogenicity strategies (35, 69) (described above). Here we have demonstrated that these phenotypic differences coincide with phylogeny and genetic differentiation and are probably the result of previously unidentified speciation events within *Histoplasma*. The identification of genetically isolated species represents a starting point to understand what genetic changes underlie medically important differences. Disease burden estimates as well as virulence studies are needed to recognize the incidence and phenotype of the diseases caused by each of the four formally described species here.

Unlike other dimorphic fungal pathogens, such as *Paracoccidioides* and *Coccidioides*, *Histoplasma* might hold the potential of producing sexual progeny in the lab. Even though this is not a trivial feat, *Emmonsiella*, the sexual stage of *Histoplasma*, can be produced in *in vitro* crosses (27, 75, 76). A possibility is to assess whether different species of *Histoplasma* can produce fertile progeny at the same rate as intraspecific crosses, which would also reveal what biological features might be involved in keeping species apart. This is of particular interest given that the species of *Histoplasma* show different levels of divergence, which in turn might represent different stages of diversification within the genus. This type of analysis might make *Histoplasma* a paradigm in the study of the evolution of reproductive isolation in ascomycetous pathogenic fungi. Of particular interest is the report that North American isolates of *Histoplasma* (likely to be NAm 1 or NAm 2) are able to produce sexual structures (cleistothecia) and mature ascospores when crossed to Africa (77). These groups are quite divergent, suggesting but not confirming that hybrid inviability might not occur readily in interspecific crosses of *Histoplasma*. This hypothesis needs to be tested with *Histoplasma* isolates of known taxonomic affinity and with recently collected isolates, as old isolates do not readily mate (75).

With the classification of different species showing strong genetic differentiation and displaying morphological and virulence differences, systematic analysis of worldwide isolate collections will be crucial to disentangle the ecology, evolution, and human incidence of each of these species. These efforts will help link how the molecular evolution of important virulence factors will ultimately impact the pathogenicity of different *Histoplasma* species and the evolution of other virulence factors.

## MATERIALS AND METHODS

### Isolate collection.

We gathered 30 *Histoplasma* isolates representing four recognized areas where histoplasmosis is endemic: 21 from North America (10 NAm 1 isolates, 11 NAm 2 isolates), 7 from Latin America (4 LAm A isolates, 3 Panama isolates), and 2 from the group Africa. Since all analyses reveal the existence of five clusters (see Results) congruent with the groups described by Kasuga et al. (31), we use the names proposed for each population by those authors: NAm 1 (North America 1), NAm 2 (North America 2), LAm A (Latin America A), Africa (Africa [*H*. *capsulatum* var. *dubosii*]), and Panama (Panama [H81 lineage]). Isolates were donated to W. Goldman during a span of 15 years. A list of donors and details for each strain is given in Table S6 at https://doi.org/10.5061/dryad.006bf. All isolates were kept in 15% glycerol at −80°C until they were ready to be subcultured and sequenced. Strains were grown in HMM (solid or liquid) at 37°C with 5% CO_2_ as described previously (5, 78). Solid medium contained 0.8% agarose (SeaKem, ME grade) and 25 mM FeSO_4_. All further analyses included only isolates for which we could obtain genomic sequences and which were available for further physiological, immunologic, and morphological experiments.

### DNA extraction.

We extracted DNA from the 30 *Histoplasma* isolates (described above). Two hundred microliters of a solution containing 1 mM EDTA, 10 mM Tris (pH 8.0), 1% SDS, 2% Triton X-100, and 100 mM NaCl was added to the cell pellet, along with 200 μl of glass beads. Cells were broken by vortexing for 2 min (split into intervals of 30 s with 30 s of incubation on ice in between), followed by incubation at 65°C for 20 min. DNA was isolated using phenol-chloroform precipitation, followed by RNase treatment with 2 μl of 2 mg/ml RNase A (Sigma, St. Louis, MO) at 37°C for 1 h and a second phenol-chloroform precipitation. The integrity of the DNA was examined by running an aliquot in a 1.8% agarose gel with a DNA mass ladder and visualized with ethidium bromide and UV light.

### Library construction.

Genomic libraries were built from high-molecular-weight DNA following the protocol described immediately above (section “DNA extraction”), using the Kappa protocol for TruSeq at the sequencing facility of the University of North Carolina, Chapel Hill. Approximately 10 µg of DNA was sonicated with a Covaris S220 to a mean fragment size of 160 bp (range, 120 to 200 bp) with the following program: 10% duty cycle at intensity 5, 100 cycles per burst, and 6 cycles of 60 s in frequency sweeping mode.

### Sequencing.

We sequenced all libraries on Illumina HiSeq 2000 machines with v3.0 chemistry following the manufacturer’s instructions. The sequencing type (single end or paired end) and coverage for each library are not shown. Libraries were pooled prior to sequencing, and 6 libraries were sequenced per lane. If all genomes were of equivalent size, then we should expect homogenous coverage across lines. We assessed the quality of the individual reads using the HiSeq Control software 2.0.5 in combination with RTA 1.17.20.0 (real-time analysis). CASAVA-1.8.2 generated and reported run statistics of each of the final FASTQ files. Resulting reads ranged from 100 to 150 bp, and the target average coverage for each line was 30×. The coverage for each isolate is not shown.

### Read mapping and variant calling.

Reads were mapped to the *Histoplasma capsulatum* genome *H. capsulatum* var. *duboisii* H88 (https://www.ncbi.nlm.nih.gov/bioproject/29163; also deposited on Dryad [https://doi.org/10.5061/dryad.006bf]), currently assembled into 16 supercontigs using bwa version 0.7.12 (79). *Histoplasma* strains vary in their karyotype and are thought to have between five and seven chromosomes, depending on the strain (80, 81). The resulting bam files were merged using Samtools version 0.1.19. Indels were identified and reads locally remapped in the merged bam files using the GATK version 3.2-2 RealignerTargetCreator and IndelRealigner functions (82, 83). Subsequently, SNPs were called using the GATK UnifiedGenotyper function with the parameter “het” set to 0.01 and all others left as default. The following filters were applied to the resulting vcf (variant call format) file: QD = 2.0, FS_filter = 60.0, MQ_filter = 30.0, MQ_Rank_Sum_filter = −12.5, and Read_Pos_Rank_Sum_filter = −8.0. Sites were excluded if the coverage was less than 5 or greater than the 99th quantile of the genomic coverage distribution for the given line or if the SNP failed to pass one of the GATK filters. Read coverage among species was compared with a one-way ANOVA followed by post hoc Tukey tests.

### Public data.

To root our trees, we obtained sequencing reads from two species of *Emmonsia*, *E. crescens* UAMH3008 and *E. parva* UAMH139, from the NCBI SRA (accession no. PRJNA178252 and PRJNA178178, respectively [84]). These species are among some of the closest relatives of *Histoplasma* (84).

### PCA.

To assess the extent of between-isolate genetic differentiation, we used principal-component analysis (PCA), a general method for summarizing high-dimensional data, as is the case of genome-wide individual genotypes. The PCA transforms a set of possibly correlated variables into a reduced set of orthogonal variables. Sampled individuals are then projected in a two-dimensional space, where the axes are the new uncorrelated variables, or principal components. Closely related individuals will form clusters, thus revealing population relationships or within-population stratification. The allele frequencies were simply the counts of each allelic class inferred from the VCF files as *Histoplasma* is haploid (49). We next calculated the proportion of genetic variance explained by each principal component (PC) and plotted two PC combinations—PC1 and PC2/PC2 and PC3/PC4—that explain the majority of the genetic variation in *Histoplasma*. All PCAs were done with the R package adegenet (85).

### LD.

We measured linkage disequilibrium (LD) within these five groups to explore the extent of recombination in *Histoplasma*. We used *r*^2^ as an LD metric, and estimated it using PLINK v.1.90b4.5. *r*^2^ ranges between 0 and 1, indicating completely independent segregation of alleles or complete linkage, respectively. For computational feasibility, we restricted our analysis to 1,000,000 randomly selected SNPs and only estimated LDs between those separated by 500 kb or less. PLINK was run with the following parameters: plink—allow-extra-chr—r2—ld-window-kb 1000—ld-window 99999—ld-window-r2 0.0. Finally we estimated the mean *r*^2^ between sites separated by physical distances, grouped in 10-bp bins (i.e., *r*^2^ between sites separated by 1 to 10 bp, 11 to 20 bp, 21 to 30 bp, etc., were averaged together).

### Phylogenetic reconstructions.

Our goal was to determine whether the genetic clusters found in *Histoplasma* satisfy the requirements to be considered phylogenetic species. We followed a phylogenetic species concept (45, 86) to recognize species, defining species as genetic clusters that are reciprocally monophyletic and for which there was genealogical concordance across genome-wide unlinked loci. We used two parallel approaches: (i) maximum likelihood (ML) trees at the genome and at the supercontig level, and (ii) Bayesian concordance analysis of the genealogies of 100-kb genomic windows.

### (i) ML phylogenetics.

To gain initial insight on the phylogenetic relationships among our sequenced isolates, and especially to explore the degree of monophyly of our putative species, we used traditional maximum likelihood (ML) phylogenetics. Reciprocal monophyly between two putative species is unlikely to be observed if they have had the chance to interbreed and exchanged genes extensively (50, 87). Given the low rates of recombination found in *Histoplasma* (31) and the high levels of linkage disequilibrium within all putative species (Fig. S1), we used whole supercontigs as unlinked loci. We focused exclusively on nuclear genomes as mitochondrial genomes have the propensity to be affected by horizontal gene transfer, thus obscuring species relationships (88, 89). We obtained whole supercontig sequences for each individual from the VCF file using the FastaAlternateReferenceMaker tool in GATK, realigned them using Mafft v.7 (90), and used them to build ML trees using RAxML version 8.2.9 (91). We inferred individual trees for each supercontig, except for 15 and 16, which were found to be completely monomorphic, as well as a “genome-wide” tree of a concatenated alignment of all supercontigs. Analyses were run under the GTR+Γ model, with 1,000 bootstrap pseudoreplicates to assess nodal support. The “genome-wide” analysis was partitioned by supercontig, with each partition having its own set of GTR+Γ model parameters, but sharing a common topology and branch lengths. All trees were rooted using *Emmonsia parva* and *E. crescens*, which are thought to have diverged from *Histoplasma* close to 100 million years ago (92).

### (ii) Bayesian concordance analyses.

To shed light on the extent of genealogical concordance at a finer scale in the genome, we performed a Bayesian concordance analysis (BCA) using BUCKy v.1.4.4 (53, 93). BUCKy estimates the level of concordance among multiple genealogies and uses it to infer the tree with the most concordant nodes, which is considered an estimate of the species tree. The level of support for each node is expressed as a concordance factor (CF), which ranges between 0 (i.e., no concordance between genealogies) and 1 (complete concordance). Therefore, this algorithm allowed us to infer the phylogenetic relationships between our putative species, while estimating the genome-wide genealogical support for their monophyly.

This analysis has two steps. First, we split the supercontig alignments into nonoverlapping 100-kb windows, and used the parallel version of MrBayes v.3.2.6 (94, 95) to generate posterior tree distributions for each one. Supercontigs shorter than 100 kb were analyzed as a single locus, and supercontigs 15 and 16 were also left out of this analysis, given their lack of genetic variation. For each window, two independent Markov chain Monte Carlo (MCMC) analyses were run for 10 million generations, sampling every 1,000, and using four chains (three heated and one cold). Likelihood estimations were done under the GTR+Γ model. Convergence was evaluated using Tracer v.1.6 (96). Runs with split frequency standard deviations above 0.1 and/or effective sample size (ESS) scores below 200 for the likelihood or posterior traces (34 of 382) were excluded from further analyses. Posterior distributions were then summarized using the mbsum tool (part of BUCKy), skipping the first 1,000 trees (10%) as burn in, to generate input files for BUCKy. Finally, we ran two independent BUCKy chains for 2 million generations (200,000 additional burn in). BUCKy uses parameter α to describe the *a priori* level of gene tree incongruence. A larger value of α corresponds to greater gene tree incongruence—the probability that 2 genes share the same topology is approximately 1/(1 + α). At the two extremes, α = 0 indicates that all genes share the same tree, while α = ∞ indicates all genes have different topologies (93). We ran our analysis using several different α values (α = 0.1, 1, 3, 5, 7, 10, and infinity) to assess whether the choice of priors affected the results and obtained nearly identical results for all values of α. Therefore, we report the results obtained using α = 5. Output files generated with other values of α for this article are available in the Dryad repository.

### Genetic distance.

To further assess the extent of genetic differentiation between phylogenetic species, we used the metric π_inter_, the average number of nucleotide differences between one sequence randomly chosen from a population and another sequence randomly chosen from a second population or species. π_inter_ followed the form π_inter *ij*_ = pairwise differences between individuals *i* and *j*/sequence length. Mean π_inter_ was the mean of all pairwise comparisons between individuals from two species. We calculated 21 mean π_inter_ values.

We also calculated π_intra_, the average pairwise genetic distance between individuals of the same species. π_intra_ followed the same form as π_inter_, but instead of calculating the average number of differences between species, it calculates the average number of differences between two randomly selected individuals of the same species. Mean π_intra_ was the mean within-species value for each of the five species. For both types of calculations, we used the function dna.dist (model=″raw") in the R package “ape” (97).

In cases where speciation is complete, π_inter_ is expected to be much larger than π_intra_. For each species pair, π_intra_ can take two values (i.e., from each of the two species), so for the pairwise comparisons π_intra_ is the pooled set of the two intraspecific distances. To compare the values of π_inter_ and π_intra_ for each species pair (10 pairwise comparisons), we used two-sample Fisher-Pitman permutation tests as implemented in the “coin” library in R (function “oneway_test”; 9,999 Monte Carlo resamplings [98]). We also calculated π_inter_ for each of the 10 pairwise comparisons and π_intra_ for the five species for each of the largest 15 supercontigs. (Supercontig 2.16 had only 4,498 bp.)

### Approximate divergence times and incomplete lineage sorting.

We calculated a rough estimate of the age of each of the phylogenetic species of *Histoplasma*, leveraging the information from our rooted phylogenetic tree. Due to the lack of an available calibration point for the *Onygenales* (i.e., fossils), we had to rely on genetic divergence and mutation rates alone. We assumed that the mutation and substitution rates in *Histoplasma* are equivalent to those measured in other fungi. The assumed substitution rate for nuclear DNA was 1 × 10^−9^ substitution/site/year (99, 100). (It is worth noting that no effort has been made to estimate the precise rate for *Histoplasma* or any other pathogenic fungus.) We converted the tree branch lengths to approximate node ages assuming a constant mutation and substitution rate.

Next, we calculated the likelihood of ancestral variation still segregating in the recipient species. We calculated the expected time for an allele segregating in the ancestral population to be either fixed (101):

$$T_{\text{fixed}}=\frac{-2N_{e}(1-p)\ln(1-p)}{p}$$

or lost (99):

$$T_{\text{lost}}=\frac{-2N_{e}p\;\ln(p)}{1-p}$$

Where *p* is the allele frequency prespeciation and *N*_*e*_ is the ancestral effective population size. We used three different values of *N*_*e*_: 10^4^, 10^5^, and 10^6^.

### Shared ancestry and gene exchange between species of *Histoplasma*. (i) TreeMix.

We looked for evidence of introgression in the nuclear genome among species of *Histoplasma* and its possible directions using the model implemented in TreeMix (102). TreeMix estimates the most likely evolutionary history of a group of populations by estimating the levels of genetic drift at a set of markers. In this case, we used genome-wide polymorphism. The analysis is done in two steps. First, it estimates the relationships between sampled populations and estimates the most likely dendrogram. Second, it compares the covariance structure modeled by this dendrogram to the actual observed covariance between populations. If a pair of populations is more closely related than expected by the strictly bifurcating tree, then the model suggests a preset number of admixture events in the history of those populations.

Since we had allele frequency data for five populations (NAm 1, NAm 2, LAm A, Panama, and Africa) and the number of inferred migrations must be lower than the number of populations, we could estimate between 0 and 4 migration events (*m*). TreeMix was run allowing for *m* = 0, 1, 2, 3, or 4. We found the most likely demographic scenario in terms of migration between species in two ways. First, we compared the log likelihood scores (LS) of each demographic event using a likelihood ratio test (LRT) with 2 df to account for changes in the number of migration edges and the direction of gene flow. The LRT follows a χ^2^ distribution and compares hierarchically nested models. Since addition of an extra migration event constitutes two additional parameters (i.e., the event itself and its direction), models with different numbers of migrations can be compared with LRTs. We did sequential LRTs to determine when addition of a new population improved the model likelihood. Second, we calculated the Akaike information criterion (AIC) for each migration scenario (103). To determine which migration scenario (i.e., number of migrations) best explained the data, we compared the AIC values for each migration model by ascribing weights to each model using weighted AIC (wAIC) with the following equation (78):

$$w_{i}=\frac{e^{\frac{{-\Delta }_{i}}{2}}}{\Sigma_{j=1}^{n}\;e^{\frac{{-\Delta }_{i}}{2}}}$$

### (ii) D statistic.

To confirm whether interspecific gene flow has indeed occurred between species of *Histoplasma* (as suggested by TreeMix [see Results]), we calculated historical levels of gene flow between three pairs of species: Africa and NAm 1, Panama and NAm 2 (both suggested by TreeMix [see below]), and NAm 1 and NAm 2, the only syntopic (i.e., cooccurring in the same geographical location) species pair of *Histoplasma* for which we had extensive sampling. We used the ABBA-BABA/D statistic (104). The ABBA-BABA test compares patterns of ancestral (A) and derived (B) alleles between four taxa. In the absence of gene flow, one expects to find equal numbers of sites for each pattern (ABBA versus BABA). However, gene flow from the third to the second population can lead to an excess of the ABBA pattern with respect to the BABA pattern; such departure from the expectation is the basis of the D statistic. As an outgroup we used *Emmonsia parva* (34, 84). Instead of sites, we used allele frequencies to obtain the ABBA and BABA counts (as described in references 55, 104, and 105). Significance was assessed using weighted block jackknifing with 50-kb windows (106). We also estimated the proportion of the genome that was introgressed with the *f*_*d*_ statistic (55). *f*_*d*_ compares the observed difference between the ABBA and BABA counts to the expected difference when the entire genome is introgressed. To test the potential influence of the choice of outgroup, we did the same calculations but instead of using *E. parva*, we used *Paracoccidioides brasiliensis*. All results were identical with this outgroup.

### (iii) Admixture.

We assessed whether there was any evidence of admixed individuals in the most extensively sampled species pair, NAm 1/NAm 2. We estimated each isolate’s admixture proportion using ADMIXTURE (107). We assumed the existence of two ancestral populations from which all observed individuals descend (*K* = 2), since we were aiming to determine the extent of admixture between isolates of two putative species, and ran independent analyses for each supercontig, to obtain information on the genomic distribution of admixture.

### Accession number(s).

All genome sequences reported in this study have been deposited in the Short Read Archive under accession numbers PRJNA416769 (samples SRX3350816–SRX3350845). Intermediary processing files (.bam and .bai) have been deposited in Dryad (https://doi.org/10.5061/dryad.006bf).
