# Supplementary figures and images for: Genome Sequences Reveal Cryptic Speciation in the Human Pathogen Histoplasma capsulatum
Source: mBio. 2017 Dec 5;8(6):e01339-17. doi: 10.1128/mBio.01339-17 (PMC5717386; doi:10.1128/mBio.01339-17)

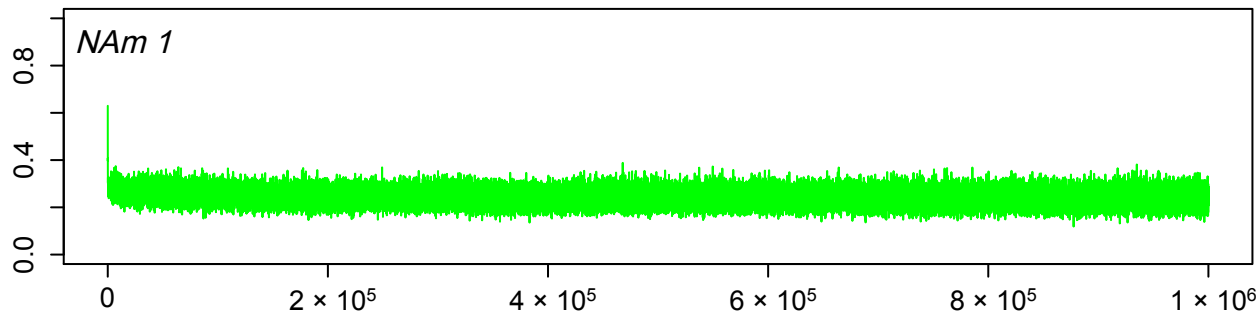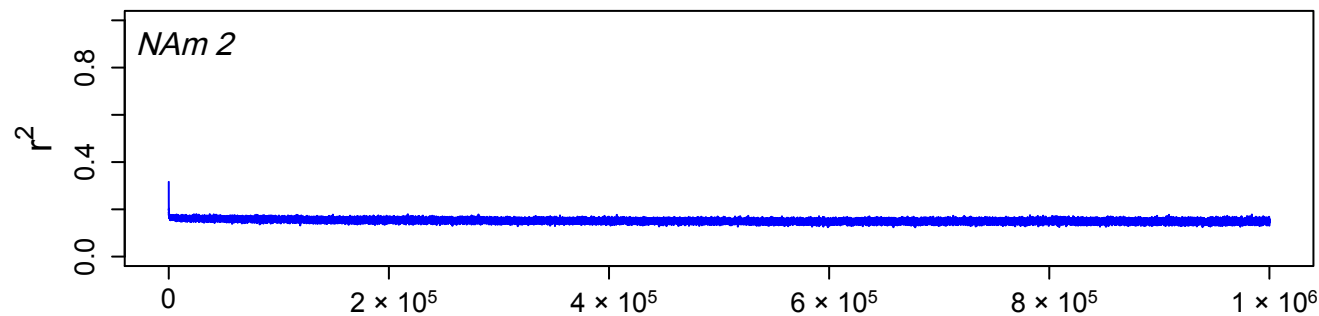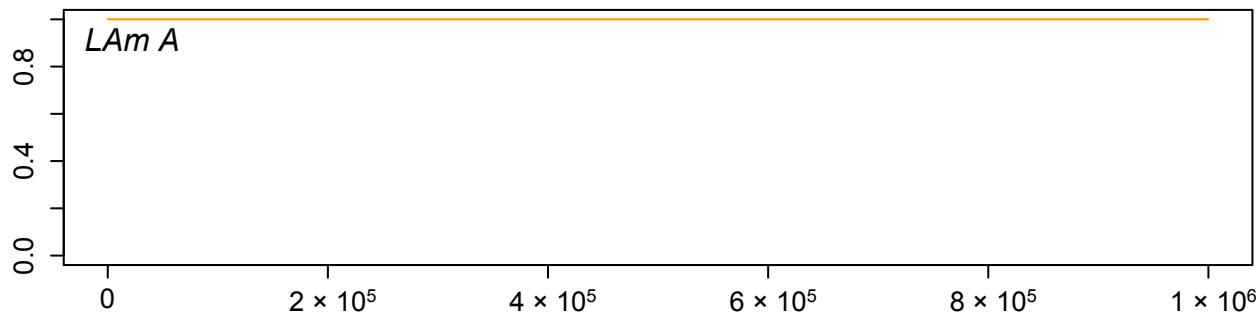

Physical distance (bp)

Supplement: FIG S1 [file mbo006173597sf1.pdf]

**A.  $N_e = 10^6$**

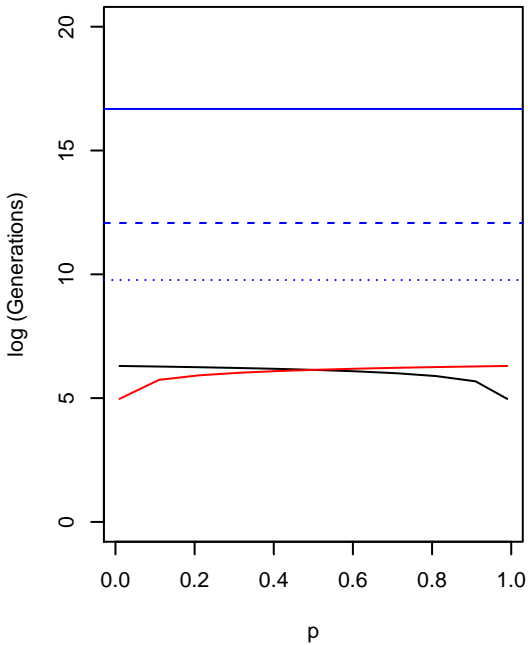

**B.  $N_e = 10^5$**

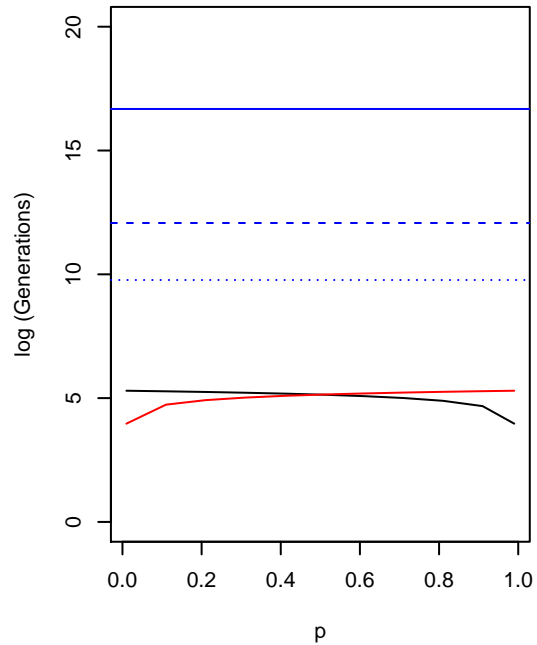

**C.  $N_e = 10^4$**

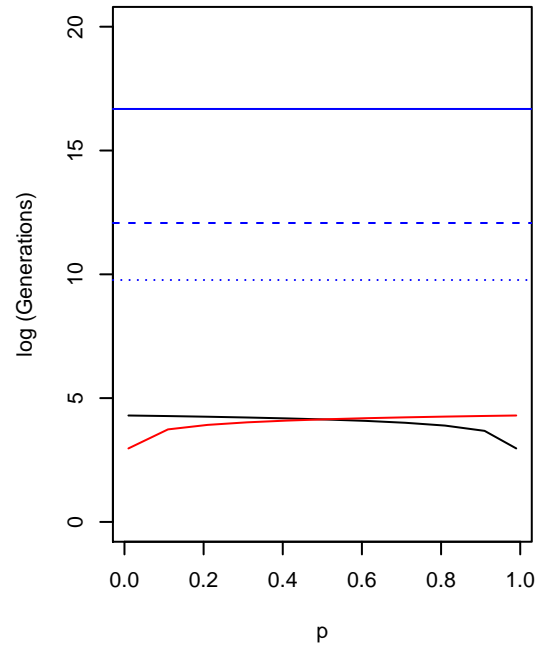

Supplement: FIG S2 [file mbo006173597sf2.pdf]
